# A retrospective study of congenital anomalies and associated risk factors among children admitted at a tertiary hospital in northwestern Tanzania

**DOI:** 10.1371/journal.pgph.0003177

**Published:** 2024-05-01

**Authors:** Wango Chaulo, Elias C. Nyanza, Moses Asori, Deborah S. K. Thomas, Florentina Mashuda

**Affiliations:** 1 Department of Environmental, Occupational and Research GIS, School of Public Health, Catholic University of Health and Allied Sciences, Mwanza, TANZANIA; 2 Department of Geography and Earth Sciences, University of North Carolina at Charlotte, Charlotte, North Carolina, United States of America; 3 Department of Pediatrics and Child Health, Bugando Medical Centre, Mwanza, TANZANIA; McGill University, CANADA

## Abstract

Congenital anomalies in Sub-Sahara Africa (SSA) are understudied despite the significant pediatric health burden. This retrospective longitudinal hospital-based study evaluated the records of 326 inpatient children under the age of two years with congenital anomalies at Bugando Medical Centre, a tertiary referral hospital in northwestern Tanzania. Classical logistic regression was used in the analysis of congenital malformation of muscles, gastrointestinal malformation, oral facial clefts, neural tube defects, and skeletal malformations. A modified poisson regression was used to model risk factors for Central Nervous System (CNS) hydrocephalus and congenital heart disease (CHD). A majority (78.8%) of children included in the study were less than six months of age. Nearly half (48.8%) were diagnosed with CHD followed by CNS hydrocephalus (10.4%) and congenital malformation of muscles (8.9%). Babies whose mothers missed periconceptual folic acid supplementation had 83% higher risk of hydrocephalus (aPR = 1.83, 95% CI = 1.11–1.96) and 78% higher for CHD (aPR = 1.78, 95% CI = 1.31–1.94). Male children had 1.67 higher odds of muscular congenital malformations (aOR = 1.67, 95% CI = 1.23–1.89). Less than 37 gestational age had a 1.86 higher odds of muscular congenital malformations (aOR = 1.86, 95% CI = 1.53–3.66). Our study highlights the critical need for folic acid supplementation and establishes a need for a registry and the potential for mapping.

## 1. Introduction

Infant mortality persists as a global health challenge [1, 2] and congenital anomalies, or birth defects, are a leading contributor to infant mortality and disability [2–4]. The incidence and burden of congenital anomalies reflects underlying healthcare inequality, nutritional deficiency, and socioeconomic inequalities. For example, it is well documented that folic acid supplementation by pregnant women reduces the risk of congenital anomalies [2, 5, 6]. Further, evidence shows that advanced maternal and paternal age, parental consanguinity (when parents are related by blood), many siblings, low birth weight, pre-term, and intrauterine infections are all associated with higher incidence of congenital anomalies [7–11]. Few studies evaluate the role of environmental exposure [12] and limited research exists in this essential public health domain in most countries in Sub-Sahara Africa (SSA), including Tanzania [13, 14], even though an estimated nine of ten children born with a serious birth defect are in low- and middle-income countries [14, 15].

Congenital anomalies affect the well-being and survival of children [4, 13, 14]. The World Health Organization (WHO) estimated more than 240,000 newborns with congenital anomalies die within the first month after delivery every year and most of those who survive fail to develop into their full age-appropriate developmental milestones [14]. Some of the most fatal congenital anomalies include congenital heart anomalies, neural tube defects, and Down’s syndrome [14, 16].

The prevalence of congenital anomalies varies substantially across the world, with developing countries bearing 94% of the burden [14, 17]. A study that investigated the regional, national, and global incident cases of congenital heart disease (CHD) between 1990 and 2017 found that at the national level, the highest CHD incidence rates were observed in developing countries in Africa and Asia, such as Central African Republic (33.8/1000), Somalia (31.9/1000), and Burundi (30.6/1000) [18]. Still, limited investigation on the burden of congenital anomalies in African countries remains a noteworthy challenge. A lack of trained professionals also contributes to underdiagnosis or possible misdiagnosis, which in turn likely leads to an underestimation of the true burden of the disease [19].

Studies have attempted to capture the prevalence of congenital anomalies in the absence of a reporting and tracking system in East Africa, and Tanzania specifically, with findings varying substantially by geographical location. The WHO estimated the number of deaths associated with congenital anomalies in Tanzania reached 9,791 or 3.32% of total deaths in 2020, ranking number 42 in the world [20]. In Dar es Salaam, 60.5 per 1,000 live births infants were reported with birth defects [4]. Similarly, a four-month hospital-based study in 2014 in the northwestern part of Tanzania found a one-time congenital anomalies prevalence of 29% among infants that were admitted [21]. In a study in Dar es Salaam, Tanzania, hydrocephalus was most commonly found (9.9 /10 000 live births respectively) [10], with a similar finding in Mwanza, Tanzania (35.9%) [21]. In another part of Lake Victoria, Entebbe, Uganda, musculoskeletal congenital anomalies, such as abdominal wall musculature, were the most common (42.7% per 1000 births) [22].

Recent studies have focused on environmental risk factors as a contributor to congenital anomalies. In the northern part of Tanzania, for example, Nyanza et al [23] reported that hydrocephalus, facial clefts, and neural tube defects were common among infants of mothers who resided in areas with artisanal and small-scale gold mining activities. This recent finding corroborates some animal model studies of teratogenesis, which found that the central nervous system, craniofacial, and neural tube development are particularly susceptible to prenatal mercury exposure [24, 25]. However, the study by Nyanza et al [20] focused only on visible congenital anomalies which likely underestimated the true burden of the disease in the area and could mislead appropriate interventions. Additionally, the role of folic acid use as a protective factor against congenital anomalies was not addressed.

Despite the recognition that congenital anomalies present a significant public health burden, they remain understudied in SSA. Further, although the protective effect of taking folic acid during pregnancy is widely accepted, the adherence in East Africa is not well documented as related to congenital anomalies. In a similar fashion, assessing geographic patterns of congenital anomalies is rarely undertaken. To address these gaps, this retrospective longitudinal hospital-based study evaluates congenital anomalies of 326 children under 2 years of age admitted July 2019-July 2022 to Bugando Medical Centre in Mwanza, Tanzania, a tertiary referral hospital serving the northwestern region. Along with modeling risk factors for types of congenital anomalies, this work geographically mapped patients’ home districts to highlight the need and possibility for a spatially explicit reporting system in Tanzania to identify possible environmental or social risk factors and to target outreach and education and support services delivery.

## 2. Materials and methods

### 2.1 Study settings

Bugando Medical Centre (BMC) is a tertiary referral hospital located in the Lake Zone in northwestern Tanzania. The hospital receives children with congenital anomalies from across the six regions of the Lake Zone area of Tanzania (Mwanza, Mara, Kagera, Geita, Shinyanga, and Simiyu) with a total population of 11,832,857 [26]. The hospital also receives patients from the Kigoma and Tabora Regions in the western and central parts of Tanzania respectively. Patients are referred to BMC from the district and regional hospitals. On average, a total of 6,200 children younger than five years of age are admitted per annum, 2000 of whom are neonates. The hospital has more than a 950-bed capacity and provides services to more than 300,000 patients and 3,800 deliveries each year.

### 2.2 Study design, study population, and sample size

We used a retrospective cross-sectional design. Data for children under the age of two years with congenital anomalies admitted to the hospital from July 1, 2019, to July 30, 2022, were extracted from electronic patient records. The electronic database was introduced in June 2019, providing an opportunity to evaluate patient records in a systematic way for congenital anomalies.

### 2.3 Inclusion and exclusion criteria

A total of 46,940 (26,472 males, and 20,472 females) medical records were reviewed and of these 338 children with a diagnosed congenital anomaly were identified for inclusion in this study. Only patients with a recorded district of residence were included. As such, 12 patient records were removed from the analysis bringing a total sample size with a definitive diagnosis of congenital anomalies to 326.

### 2.4 Data collection

A data collection checklist (see *S1 Checklist*) was used to extract information from patient archived files for children younger than two years of age from an electronic records management system. The initial search included ‘congenital anomaly’ or ‘congenital malformation’ or ‘birth defect’. Each record was subsequently reviewed by one pediatrician trained in medical genetics (FM) and one medical doctor (SJ) for categorization into central nervous system (CNS) hydrocephalus, congenital heart diseases (CHDs), gastrointestinal, oral facial clefts, skeletal malformations, or malformations of the muscles. Along with the diagnosis, patient information was extracted from the electronic record, including age, sex, referral status, referral diagnosis, date of admission, birth weight, management given, and outcome (discharged, referred, or died) (*see S1 Data*). Information on critically ill infants, including those admitted with imminent risk of death like deteriorated vital signs, unconsciousness, and gasping, were included.

### 2.5 Statistical analysis

Data cleaning and analysis were performed using *STATA version 15 (Stata Corp LP)* [27]. A preliminary exploration of the data was done to check for missing values, duplicates, and unusual observations before analysis. Specific congenital anomalies were grouped based on the International Statistical Classification of Diseases and Related Health Problems 10th Revision (ICD10) to increase the power of the groups, including congenital heart diseases (CHD), central nervous system (CNS) hydrocephalus, oral facial clefts, gastrointestinal malformations, skeletal malformations, and malformations of the muscles (*see S1 Checklist*).

Continuous variables were summarized using median and interquartile range while categorical variables were summarized using frequency and percentages. Classical logistic regression was used to determine the association between the congenital malformation of muscles, gastrointestinal malformation, oral facial clefts, neural tube defects, and skeletal malformations with age, maternal folic acid supplementation, birthweight of the children, gestation age, age of the children, maternal education, and parity. However, since classical logistic regression has the tendency to overestimate the true risk when the population prevalence is >10, we used modified poisson regression to model risk factors of CNS hydrocephalus and CHD due to their high higher prevalence. All independent variables with *p-*value <0.05 in the bivariate analysis were entered into a multivariable model to adjust for the confounding effects. Independent variables with *p-*value <0.05 in multivariable analysis were considered statistically significantly associated with the outcomes of interests.

### 2.6 Geographic mapping

Because hospital-based data do not represent all disease in a population, we undertook descriptive mapping to illustrate the need for a regional reporting system for congenital anomalies. Maps were produced using *ArcGIS Desktop 10*.*3* [28]. Base maps and population numbers were obtained from the Tanzania National Bureau of Statistics [29, 30]. Although not representing prevalence or incidence, it does provide insights into locations where patients might need care, gaps in seeking care, higher than expected types of congenital anomalies, or where certain risk factors might drive higher than expected rates. While not entirely systematic, visualization can geographically guide further investigation, intervention, or education targeted towards particular communities.

### 2.7 Ethics consideration and confidentiality

Ethical clearance was sought from the Co-Joint Catholic University of Health and Allied Sciences (CUHAS) and Bugando Medical Center (BMC) Research and Ethics Review Committee (CREC) (*Ref*: *CREC/553/2022*). Administrative approval was obtained from relevant authorities at Bugando Medical Center (*Ref*: *AB/286/317/01*). The present study used secondary data archived from electronic patient records. Therefore, we did not have direct contact with involved patients. Consent forms for caregivers/parents and assent forms for children under the age of two years were waived by the Ethics Review committee (i.e., CREC). Data of children under the age of two years with congenital anomalies admitted to the hospital from July 1, 2019, to July 30, 2022, were extracted from electronic patient records (*see S1 Data*).

## 3. Results

### 3.1 Background characteristics of the study participants

Characteristics of the 326 children are shown in Table 1. A majority (78.8%) were less than six months. The median age was two (2) months (IQR: 1–5). More than half (53.7%) of the children were male. A majority (56.3%) came from Mwanza Region, particularly from Ilemela District (24.5%). Table 2 presents the types of congenital anomalies. The majority (48.8%) of the children were diagnosed with congenital heart diseases and 10.4% with CNS hydrocephalus. In addition, 8.9% were diagnosed with congenital malformation of muscles and 7.7% with gastro-intestinal malformations.

**Table 1:** Characteristics of the study participants (N = 326).

Variables	N	%
Age in months		
less than six months	257	78.8
six months and above	69	21.2
Median age (IQR)	2(1–5)*	-
Sex of the child		
Male	164	50.3
Female	162	49.7
Education level of guardians		
Primary education	209	64.1
Secondary education	108	33.1
College education	9	2.8
Gestation age (weeks)		
Below 37	250	76.7
37 and above	76	23.3
Birth weight		
Very low birth weight	4	9.2
Low birth weight	98	30.1
Normal weight	198	60.7
Folic acid supplementation		
Yes	120	36.8
No	206	63.2
Parity		
Less than three	19	5.8
Three and above	22	6.8
Missing	285	87.4
Outcomes		
Repaired	36	11.1
Died	5	1.5
Unknown	285	87.4

*IQR = *Interquartile Range; age is in months*

**Table 2:** Distribution of congenital anomalies among patients admitted at Bugando Medical Centre.

Variables	N	%
Congenital heart diseases	159	48.8
CNS hydrocephalus	34	10.4
Congenital malformation of muscles	29	8.9
Gastro-intestinal malformations	39	12
Oral facial clefts	20	6.1
Neural tube defects	15	4.6
Skeletal malformations	15	4.6
Others congenital anomalies	15	4.6

### 3.2 Spatial distribution of the relative risk of overall congenital anomalies

**Fig 1:**
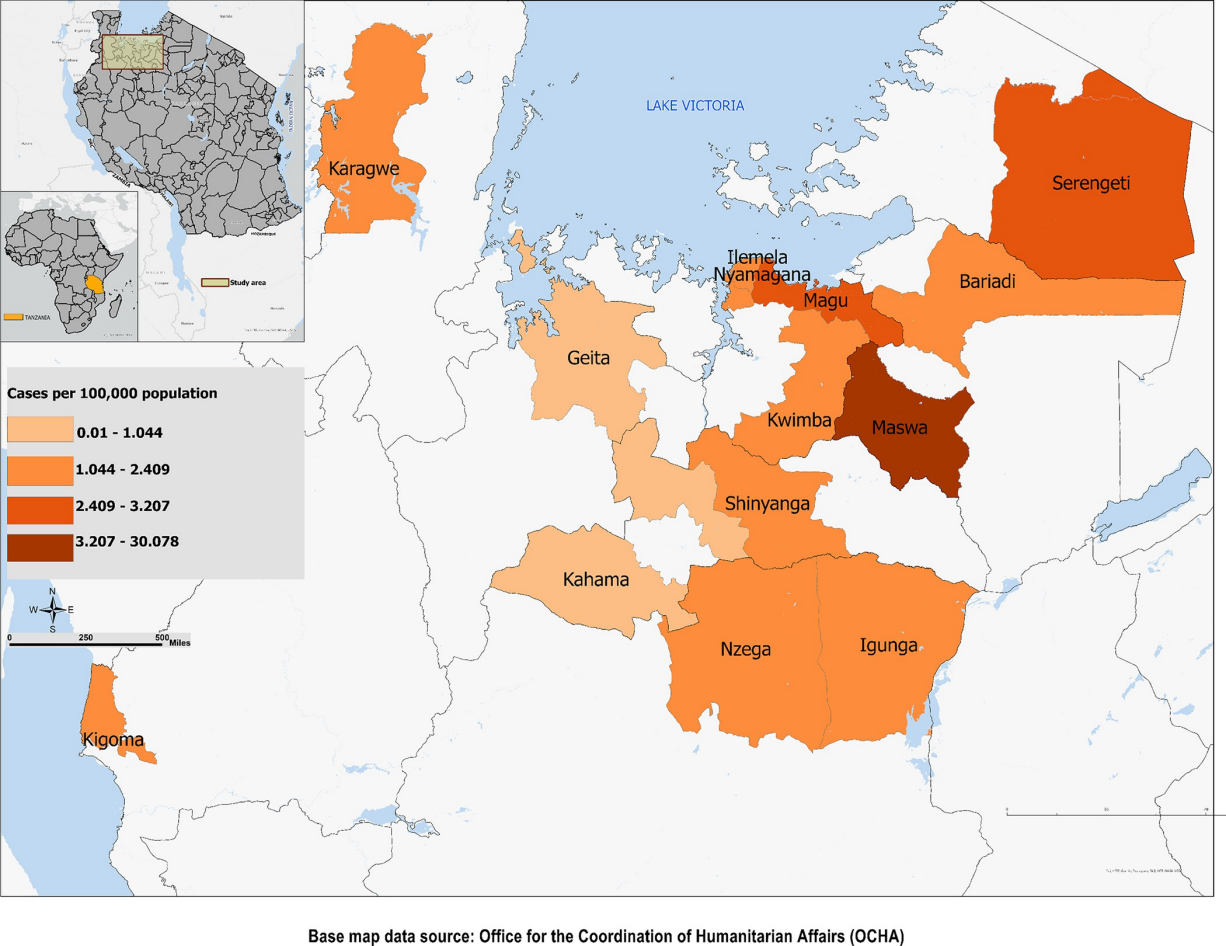
Spatial distribution of congenital anomalies (cases per 100,000). Base map data source: OCHA, https://data.humdata.org/dataset/cod-ab-tza. Source to terms of use: https://data.humdata.org/faqs/terms. Map created by: Moses Asori and Deborah Thomas.

Fig 1 presents the spatial distribution of the adjusted cases (cases per 100,000 population) of diagnosed congenital anomalies in the Lake Zone of Tanzania based on the zonal referral hospital records at Bugando Medical Centre. Maswa District had the highest cases (6.65–30.07 per 100,000) for congenital anomalies followed by Serengeti District (2.02–6.65 per 100,000) (Table 3). Ilemela, Magu, Nzega, Karagwe, Shinyanga, Nyamagana, Igunga, Kigoma, Kwimba, and Bariadi Districts had between 1.10 and 2.02 cases per 100,000 population. However, Geita and Kahama Districts had the lowest with a range of 0.01–1.10 per 100,000 population, although these districts are some of the furthest away. Other equally far districts had no patients admitted to the regional hospital. Generally, there was no concrete pattern of highly prevalent areas. However, there were a number of districts with no data and many others with small numbers and so spatial analysis was not possible.

**Table 3:** Normalized congenital anomalies prevalence rates per 100,000 population for respective regions and districts in the Lake Zone.

Variables	Region population*	Congenital Anomalies	
***Region***		**n**	Pr**
Geita	1,739,530	13	0.75
Kagera	2,458,023	19	0.77
Kigoma	2,127,930	11	0.52
Mara	1,743,830	28	1.6
Mwanza	2,772,509	183	6.6
Shinyanga	1,534,808	27	1.8
Simiyu	1,584,157	23	1.40
Tabora	2,291,623	17	0.74
***District***		**n**	Pr**
Bariadi	422,916	8	1.9
Ilemela	343,001	77	2.3
Bunda	335,061	19	2.4
Nyamagana	363,452	71	2.2
Bunda	335,061	19	2.4
Shinyanga	495,808	12	1.6
Nzega	417,097	9	1.9
Magu	299,759	8	2.7
Serengeti	249,420	8	3.2
Maswa	26,597	6	30.1
Geita	807,619	11	1.0
Igunga	399,727	7	2.0
Kahama	766,010	5	1.0
Karagwe	332,020	11	2.4
Kigoma	427,024	9	1.9
Kwimba	406,509	18	2.0

*Estimated Population based on 2012 TZS Census Report; **Pr = Prevalence rate

### 3.3 Congenital heart diseases (CHDs)

Babies born to mothers who did not take folic acid supplementation were more likely to be diagnosed with CHDs (cPR = 1.71, 95% CI = 1.10–1.98). Children with a very low birth weight (cPR = 1.75, 95% CI = 1.11–1.97) and low birth weight (cPR = 1.71, 95% CI = 1.39–2.11) were more likely to be diagnosed with CHDs (Table 4). In the adjusted model, low birth weight (aPR = 1.78, 95% CI = 1.37–3.52), very low birth weight (aPR = 1.73, 95% CI = 1.03–1.94), and no folic acid supplementation (aPR = 1.78, 95% CI = 1.31–1.94) remained statistically significantly associated with the risk of CHDs (Table 5).

**Table 4:** Unadjusted associations between different types of congenital anomalies and their associated predictors.

Variables	*Congenital heart diseases*	*CNS hydrocephalus*	*Congenital malformation of muscles*	*Gastro-intestinal malformations*	*Oral facial clefts*	*Neural tube defects*	*Skeletal malformations*
	cPR(95%CI)	cPR(95%CI)	cOR(95%CI)	cOR(95%CI)	cOR(95%CI)	cOR(95%CI)	cOR(95%CI)
*Age in months*							
<6 months	0.71(0.56,1.88)	0.34(0.24,1.76)	0.67(0.34,1.89)	1.67(1.04,1.86)*	1.24(0.22,1.56)	0.56(011,1.54)	1.51(1.23,1.98)*
≤6 months	1	1	1	1	1	1	1
*Sex of the child*							
Male	1.0(0.80,1.25)	1.34(0.54,1.67)	1.25(1.07,1.63)*	1.76(1.21,2.72)*	1.67(0.45,1.76)	1.67(0.31,1.89)	0.67(0.33,1.83)
Female	1	1	1	1	1	1	1
*Education level of guardians*							
Primary	1.13(0.56,1.78)	1.34(0.65,1.98)	1.18(0.42,1.89)	0.45(0.67,1.80)	1.43(0.33,1.56)	0.98(0.13,1.64)	0.43(0.33, 1.53)
Secondary	1.16(0.92,1.45)	1.56(0.67,1.79)	1.34(0.76,1.45)	1.64(0.52,1.96)	1.56(0.43,1.56)	0.74(0.24,0.88)*	0.57(0.43,1.50)
College	1	1	1	1	1	1	1
*Gestation age (weeks)*							
<37	1.04(0.79,1.36)	1.56(0.09,1.96)	1.97(1.56,3.89)*	1.63(0.48,1.82)	1.75(0.56,3.12)	1.56(1.25,2.89)*	1.79(1.56, 3.12)*
≥37	1	1	1	1	1	1	1
*Birth weight*							
Very LBW	1.75(1.11,1.97)*	1.96(1.04,1.97)*	1.67(1.43,3.78)*	2.03(1.31,3.67)*	1.67(1.21,3.98)*	1.82(1.52,1.96)*	1.68(1.11, 3.76)*
LBW	1.71(1.39,2.11)**	1.78(1.23,2.11)*	1.67(1.43,2.67)**	1.54(1.08,2.74)**	1.54(0.43,2.87)	1.78(1.34,2.67)*	0.54(0.21,2.03)
Normal weight	1	1	1	1	1	1	1
*Folic acid supplementation*							
Yes	1	1	1	1	1	1	1
No	1.71(1.10,1.98)*	1.87(1.56,1.88)*	1.56(0.43,1.76)	1.78(0.90,1.89)	1.75(1.43,2.57)*	1.76(1.47,1.98)*	1.78(1.12,2.98)*
Parity							
<3	1	1	1	1	1	1	1
≥3	0.65(0.13,1.43)	0.75(0.03,2.44)	1.54(1.03,2.67)*	0.77(0.61,1.97)	1.61(0.22,1.82)	0.76(0.16,1.97)	1.77(1.36,1.94)*

CI, confidence interval, cPR = crude prevalence ratio; Cor = crude odds ratio, *p<0.05

**p<0.001

**Table 5:** Adjusted association between congenital anomalies and risk factors.

Variables	*Congenital heart diseases*	*CNS hydrocephalus*	*Congenital malformation of muscles*	*Gastro-intestinal malformations*	*Oral facial clefts*	*Neural tube defects*	*Skeletal malformations*
	aPR(95%CI)	aPR(95%CI)	aOR(95%CI)	aOR(95%CI)	aOR(95%CI)	aOR(95%CI)	aOR(95%CI)
***Age in months***							
<6 months	0.89(0.52,2.89)	0.45(0.02,1.87)	0.67(0.23,2.65)	1.87(1.31,1.96)*	1.75(0.42,2.44)	0.47(0.39,1.88)	1.87(1.43,2.89)*
≤6 months	1	1	1	1	1	1	1
***Sex of the child***							
Male	1.21(0.40,1.14)	1.34(0.12,1.45)	1.67(1.23,1.89)*	1.59(0.34,1.97)	1.33(0.67,1.45)	1.67(0.13,1.76)	1.37(0.43,1.86)
Female	1	1	1	1	1	1	1
***Education level of guardians***							
Primary	1.24(0.54,1.67)	1.43(0.32,1.89)	1.45(0.34,1.78)	1.43(0.19,1.95)	1.02(0.05,1.56)	0.54(0.27,0.76)*	0.02(0.05,1.56)
Secondary	1.27(0.93,1.67)	1.34(0.04,1.78)	1.59(0.78,1.34)	1.97(0.37,1.58)	1.67(0.65,1.89)	0.87(0.14,1.99)	0.69(0.65,1.86)
College	1	1	1	1	1	1	1
***Gestation age (weeks)***							
<37	1.84(0.95,3.57)	1.63(1.25,3.98)*	1.87(1.56,3.66)*	1.78(1.45,3.89)*	1.43(1.21,3.78)	1.90(1.34,3.56)*	1.56(1.22,3.45)*
≥37	1	1	1	1	1	1	1
***Birth weight***							
Very LBW	1.73(1.03,1.94)*	1.91(1.12,1.98)*	1.67(1.23,1.99)*	1.56(1.03,1.97)*	1.78(1.12,1.89)*	1.87(1.43,1.96)*	1.79(1.23,1.86*)
LBW	1.78(1.37,3.52)*	1.56(1.23,3.78)*	1.75(1.43,3.57)*	1.43(1.17,3.78)*	1.87(0.34,2.65)	1.74(1.33,3.67)*	1.32(0.31,2.78)
Normal weight	1	1	1	1	1	1	1
***Folic acid supplementation***							
Yes	1	1	1	1	1	1	1
No	1.78(1.31,1.94)*	1.83(1.11,1.96)*	0.78(0.54,1.71)	0.69(0.26,1.90)	1.95(1.54,4.67)*	1.91(1.34,2.89)*	1.67(1.13,4.69)*
Parity							
<3	1	1		1		1	
≥3	0.65(0.13,1.43)	1.40(0.11,1.82)	1.87(0.41,1.93)	1.83(0.03,1.97)	1.36(0.09,1.75)	1.89(1.23,2.97)*	1.59(1.06,1.85)*

CI, confidence interval, cPR = crude prevalence ratio; Cor = crude odds ratio

*p<0.05

**p<0.001

### 3.4 CNS hydrocephalus

Children with very low birth weight (cPR = 1.96, 95% CI = 1.04–1.97) and low birth weight (cPR = 1.78, 95% CI = 1.39–2.11) were more likely to be diagnosed with CNS hydrocephalus (Table 4). Babies born to mothers who were not supplemented with folic acid were also more likely to be diagnosed with CNS hydrocephalus (cPR = 1.87, 95% CI = 1.56–1.88). In the adjusted model, children with less than 37 weeks gestational age (aPR = 1.63, 95% CI = 1.25–3.98), children with low birth weight (aPR = 1.56, 95% CI = 1.23–3.78), and children with very low birth weight (aPR = 1.91, 95% CI = 1.12–1.98) were more likely to be diagnosed with hydrocephalus (Table 5).

### 3.5 Malformations of muscles

Male children had 1.25 higher odds of being diagnosed with congenital malformations of muscles (cOR = 1.25, 95% CI = 1.07–1.63) (Table 4). Children with low birth weight (cOR = 1.67, 95% CI = 1.43–2.67), very low birth weight (cOR = 1.67, 95% CI = 1.43–3.78), and less than 37 weeks gestational age (cOR = 1.97, 95% CI = 1.56–3.89) were more likely diagnosed with malformations of the muscles. In the adjusted model, babies below 37 gestation age had 1.86 higher odds a congenital malformations of muscles diagnosis (aOR = 1.86, 95% CI = 1.53–3.66) (Table 5). Male children had 1.67 higher odds of being diagnosed with congenital malformations of muscles (aOR = 1.67, 95% CI = 1.23–1.89).

### 3.6 Gastrointestinal malformations

In the crude analysis, children with very low birth weight had 2.03 higher odds of being diagnosed with gastrointestinal malformations (cOR = 2.03, 95% CI = 1.31–3.67) and those with low birth weight had 1.54 higher odds (cOR = 1.54, 95% CI = 1.08–2.74) of this diagnosis (Table 4). Male children also had 1.76 higher odds of being diagnosed with gastrointestinal malformations (cOR = 1.76, 95% CI = 1.21–2.72). Those who were born to mothers who were not supplemented with folic acid were diagnoses at a 1.78 higher rate for gastrointestinal malformations (cOR = 1.78, 95% CI: 0.90–1.89). In the adjusted model (Table 5), babies with very low birth weight had 1.56 higher odds of being diagnosed with gastrointestinal malformations (aOR = 1.56, 95% CI = 1.03–1.97) and male participants had 1.78 higher odds of being diagnosed with gastrointestinal malformations (aOR = 1.78, 95%CI = 1.14–1.86) Children born at a gestation age of less than 37 weeks had 1.78 higher odds (aOR = 1.78, 95% CI = 1.45–3.89) of being diagnosed with gastrointestinal malformations compared to those at a gestation age of ≥37 weeks.

### 3.7 Oral facial clefts

In the unadjusted model (Table 4), children with very low birth weight had 1.67 higher odds of being diagnosed with oral facial clefts (cOR = 1.67, 95% CI = 1.21–3.98). Children born to mothers who were not supplemented with folic acid had 1.75 higher odds of being diagnosed with oral facial clefts (cOR = 1.75, 95%CI = 1.43–2.57). In the adjusted model (Table 5), children with very low birth weight had 1.78 higher odds of being diagnosed with oral facial clefts (aOR = 1.78, 95% CI = 1.12–1.89). Children born from mothers who were not supplemented with folic acid had 1.95 higher odds of being diagnosed with oral facial clefts (aOR = 1.95, 95%CI = 1.54–4.67).

### 3.8 Neural tube defects

In the crude analysis (Table 4), children born less than 37 weeks gestational age had 1.56 higher odds of being diagnosed with neural tube defects (cOR = 1.56, 95% CI = 1.25–2.89). Those with very low birth weight had 1.82 higher odds of being diagnosed with neural tube defects (cOR = 1.82, 1.95CI = 1.52–1.96) and low birthweight had a 1.78 higher odds (cOR = 1.78, 95% CI-1.34–2.67). Those not supplemented with folic acid had 1.76 higher odds of diagnosis (cOR = 1.76, 95% CI = 1.47–1.98). Secondary education reduced the potential for diagnosis (cOR = 0.74, 95% CI = 0.74–0.88). In the adjusted model (Table 5), babies below 37 gestational age had 1.90 higher odds of being diagnosed with neural tube defects (aOR = 1.90, 95% CI = 1.34–3.56). Those with very low birth weight had 1.87 higher odds of being diagnosed with neural tube defects (aOR = 1.87, 95%CI = 1.43–1.96).

### 3.9 Skeletal malformations

In the unadjusted model (Table 4), children with very low birth weight had a 1.68 higher odds of being diagnosed with skeletal malformations (cOR = 1.68, 95% CI = 1.11–3.76). Babies who were born from mothers who were not supplemented with folic acid had 1.78 higher odds of being diagnosed with skeletal malformation (cOR = 1.78, 95%CI = 1.12–2.89). Children born from mothers with ≥3 parity had 1.77 higher odds of being diagnosed with skeletal malformation (cOR = 1.77, 95% CI = 1.36,1.94). In the adjusted model (Table 5), children with very low birth weight had 1.79 higher odds of being diagnosed with oral facial clefts (aOR = 1.79 95% CI = 1.23–1.86). Mothers being not supplemented with folic acid had 1.67 higher odds of having a baby diagnosed with skeletal malformations (aOR = 1.67, 95%CI = 1.13–4.69). Children whose mothers had ≥3 parity had increased odds of being diagnosed with skeletal malformations (aOR = 1.59, 95% CI = 1.06–1.85).

## 4. Discussion

Genetic factors, advanced maternal and paternal age, parental consanguinity, increasing number of siblings/children, preterm birth, low birth weight, maternal malnutrition (such as iodine deficiency and folic acid deficiency), and intrauterine infections, all have a role in congenital anomalies formation, contributing to about 60% of the cases globally [7, 9, 10]. The current study documents an increased likelihood of being diagnosed with congenital anomalies for infants born with low birth weight, which is consistent with previous studies also showing that very low birth weight and low birth weight were associated with increased incidence of congenital anomalies [9, 10, 31–36]. Further, previous studies have reported that one third of low birth weight infants with an intraventricular hemorrhage had posthemorrhagic hydrocephalus [31, 34–36]. While the current study did not capture information about intraventricular hemorrhage, it did document an almost twofold increase in being diagnosed with CNS hydrocephalus for low birth weight infants. Additional research to document the possible causal pathways between low birth weight, preterm birth, and incidence of congenital anomalies is needed to enhance interventions that address low birth weight, preterm birth and congenital anomalies.

Among the individual children with congenital anomalies admitted at BMC in northwestern Tanzania, most children were less than 6 months of age, reinforcing the need for parents to seek early care. CHDs were the most common type of congenital anomalies admitted at BMC followed by hydrocephalus and congenital muscles malformations. Eight years ago, spina bifida, hydrocephalus, and musculoskeletal and gastrointestinal malformations were the leading forms of pediatric congenital anomalies in Tanzania [21]. Our current findings corroborate the study of Thejaswini et al., where CHD and hydrocephalus were the most common forms of congenital anomalies among Indian infants admitted at a tertiary hospital [16].

The role of maternal nutrition is clearly of upmost importance. In our study, the odds of being diagnosed with congenital anomalies, including CHDs, NTD, oral facial clefts (OFCs), and skeletal malformations (SMs), were all strongly associated with a lack of periconceptional folic acid supplementation. This is consistent with a previous study of 445 women in Mwanza, Tanzania who had infants with congenital anomalies with only 15% having used folic acid supplementation during the first trimester of pregnancy [21]. Taking folic acid supplementation prior to conception can reduce incidence of neural tube defects by 73% [5, 6]. Our current study has a similar finding with most of the mothers (63.2%) not having taken folic acid supplementation. It is crucial to provide folic acid supplementation at antenatal care clinics and for programs to educate and to encourage pregnant women to adhere to the regular intake of periconceptional folic acid supplementation [23].

Male children were more often diagnosed with skeletal muscles and gastrointestinal malformations than females. Even though the mechanism is not well understood previous studies have documented sex-linked genetic inheritance where parents with recessive congenital anomaly traits are more likely to transfer to their male children [8]. Since these gender-related findings are limited in scope, more research is needed. For example, a systematic review indicated that gender-specific susceptibility to neurotoxins has not been given adequate attention [37] and so understanding the role of neurotoxins is an important avenue of further investigation.

Evidence is mounting that gene-environment interaction could also play a role, with estimations that around 25% of congenital anomalies are due to the convoluted interaction between genetic factors and the physical environment [38]. For example, being in gold mining areas elevates children’s propensity to be diagnosed with congenital anomalies due to potentially elevated exposure to toxic chemical substances [24]. A recent large prospective longitudinal study with 1078 pregnant women study in areas with high environmental exposure reported a two-fold increase in the risk of visible congenital anomalies for every 10-fold increase in prenatal total mercury blood concentrations [23]. Another study from China reported 8.8 odds of neural tube defects among infants exposed to mercury prenatally as compared to a control group [39].

In the present study, children being admitted for CNS hydrocephalus from more remote districts were most commonly from Geita and Kigoma Districts. Given the distance, the small numbers likely means that there are many more cases than represented in regional hospital records. These areas have concentrations of artisanal and small-scale rudimentary gold mining using mercury amalgamation, which contaminates the environment and affects worker’s health [39, 40]. Evidence from animal model studies of teratogenesis supports the argument that the central nervous system, craniofacial, and neural tube development are particularly susceptible to prenatal exposure to toxic chemical elements such as mercury [24, 25]. As such, it is possible that environmental exposures contribute to this increase, even though this only represents those children who present at hospital across the region.

There is a wide range of neuro-toxicants of public health concerns, such as heavy metals, anticonvulsant pharmaceuticals, organochlorine pesticides, polychlorinated biphenyls (PCBs), toluene, tetrachloroethylene among others which are known to injure the developing fetus and could be contributing to the rise in prevalence of birth defects [12, 23–25, 41]. Having a systematic reporting system would enable investigating multidimensional risk factors to prevent congenital anomalies. While the descriptive mapping in the current study could not be used to establish spatial variation in prevalence or incidence, a reporting system with more complete data on congenital anomalies would further the ability to understand geographic (social, environmental, genetic, or health access) risk factors to inform targeting educational and environmental programs and ensure distribution of care.

### 4.1 Limitations of the study

The present study used hospital-based data and so we were unable to establish prevalence across the region or conduct spatial analysis that could reveal risk factors to target interventions. Archived patient records were collected for patient management purposes and not for research. As a result, some of the socio-demographic and socio-economic data were missing, limiting the ability to explore all the possible risk factors. Because the current study focused on retrospective data at a tertiary referral hospital, this study undoubtably underestimated the true prevalence of congenital anomalies cases and cannot be generalized to other regions. Many families cannot or do not access the largest and most sophisticated hospital in the area due to possible stigmatization, cultural norms, and beliefs, and/or socio-economic challenges associated with expenses for travel, treatment, and hospital stay, as the majority in these settings are of low socio-economic status and rural [23]. Further, health professionals outside of the tertiary hospital are not as highly trained in pediatric medicine, likely also contributing to underreporting.

## 5. Conclusion

Congenital anomalies in the Lake Zone of Northern Tanzania are a silent neglected public health issue affecting maternal and child health. Our study showed that maternal the lack of periconceptual folic acid supplementation, shortened gestational age, male sex of the child, and low birth weight increased the likelihood of being diagnosed with congenital anomalies. Our findings provide initial evidence to develop and scale up existing programs to ensure pregnant women have easy access to folic acid and receive the necessary care to increase the birth weight of children. The results combined with the mapping illustrate the need for a robust congenital anomaly reporting system.

## Supporting information

S1 Checklist Checklist for extracting information for children with congenital anomalies. (DOCX)

S1 Data De-identified data and detailed information regarding the participants. (CSV)
