# Supplementary material for: A retrospective study of congenital anomalies and associated risk factors among children admitted at a tertiary hospital in northwestern Tanzania
Source: PLOS Glob Public Health. 2024 May 1;4(5):e0003177. doi: 10.1371/journal.pgph.0003177 (PMC11062536; doi:10.1371/journal.pgph.0003177)
Supplement: S1 Checklist — (DOCX) [file pgph.0003177.s001.docx]

***S1 Checklist****:* Checklist for extracting information for children with congenital anomalies

| **Patient ID:** | | | **Name of the Hospital** | | | | **Date of Admission**  **……./……./………** | |
| --- | --- | --- | --- | --- | --- | --- | --- | --- |
| Age  (Months) | Residence | | Sex | Birth  Weight: | Type of Pregnancy | Gestation  Age (Weeks) | Type of Anomaly | Outcomes |
|  | Village | District |  |  |  |  |  |  |
| Parity | Gravidity | Age (Mother) | Maternal  Occupation | Maternal Education | Maternal Health History | Maternal Risk Behavior | |  |
| **Management Notes** | | | | | | | | |
| **List of Congenital Anomalies Extracted from the Electronic Record System**  The initial search included ‘congenital anomaly’ or ‘congenital malformation’ and then each record was subsequently reviewed and classified as follows:   1. **Central Nervous System (CNS) Hydrocephalus*:*** *(a) spina bifida, (b) hydrocephalus, (c) anencephaly, (d) meningocele, (e) meningomyocele, (f) microcephaly (g) agenesis of cerellar vermis (h) enchephalocele (i) craniorachischisis* 2. **Congenital heart diseases (CHDs):** *(a) congenital heart disease, (b) atrial septal defect, (c) aortic atresia (d) patent ductus arteriosus (e) single umblical artery (f) single ventricle (g) hypoplastic left heart (h) pulmonary artery hypertension (i) skeletal system (j) skeletal dysplasia (k) sacral agenesis (l) polydactyly* 3. **Gastrointestinal:** *(a) imperforate Anus, (b) esophageal (c) esophageal asresia (d) intraperitoneal cyst* 4. **Oral facial clefts:** *(a) cleft lip (b) cleft palate* 5. **Skeletal malformations:** *(a) talipes* 6. **Malformations of the muscles:** *(a) umbilical hernia, (b) gastroschisis, (c) omphaloecele* | | | | | | | | |
| ***International Classification of Diseases and Related Health Problems 10th Revision (ICD10)***   1. **Neural tube defects included:** *Spina bifida (a)meningomyelocele and (b)menongocelle, and encephalocelle (occipital encephocelle).* 2. **Congenital heart diseases (CHDs):** *patent ductus arteriosus, atrial septal defect, ventricular septal defect, and congenital malformations of cardiac septa.* 3. **Gastrointestinal malformations:** *Duodenal atresia, and anal rectal malformations* 4. **Oral facial clefts:** *cleft lip and cleft palate.* 5. **Skeletal malformations:** *Tallipes (varus/valgus).* 6. **Malformations of the muscles:** *Omphalocele, umbilical hernia, inguinal hernia, scrotal swelling, and congenital hernia.* | | | | | | | | |
